# Development of Real-Time PCR Assay to Specifically Detect 22 *Bifidobacterium* Species and Subspecies Using Comparative Genomics

**DOI:** 10.3389/fmicb.2020.02087

**Published:** 2020-08-28

**Authors:** Hyeon-Be Kim, Eiseul Kim, Seung-Min Yang, Shinyoung Lee, Mi-Ju Kim, Hae-Yeong Kim

**Affiliations:** Department of Food Science and Biotechnology, Institute of Life Sciences and Resources, Kyung Hee University, Yongin, South Korea

**Keywords:** *Bifidobacterium*, real-time PCR, pan-genome, whole-genome sequence, probiotic, comparative genomics, identification, detection method

## Abstract

*Bifidobacterium* species are used as probiotics to provide beneficial effects to humans. These effects are specific to some species or subspecies of *Bifidobacterium*. However, some *Bifidobacterium* species or subspecies are not distinguished because similarity of 16S rRNA and housekeeping gene sequences within *Bifidobacterium* species is very high. In this study, we developed a real-time polymerase chain reaction (PCR) assay to rapidly and accurately detect 22 *Bifidobacterium* species by selecting genetic markers using comparative genomic analysis. A total of 210 *Bifidobacterium* genome sequences were compared to select species- or subspecies-specific genetic markers. A phylogenetic tree based on pan-genomes generated clusters according to *Bifidobacterium* species or subspecies except that two strains were not grouped with their subspecies. Based on pan-genomes constructed, species- or subspecies-specific genetic markers were selected. The specificity of these markers was confirmed by aligning these genes against 210 genome sequences. Real-time PCR could detect 22 *Bifidobacterium* specifically. We constructed the criterion for quantification by standard curves. To further test the developed assay for commercial food products, we monitored 26 probiotic products and 7 dairy products. Real-time PCR results and labeling data were then compared. Most of these products (21/33, 63.6%) were consistent with their label claims. Some products labeled at species level only can be detected up to subspecies level through our developed assay.

## Introduction

Probiotics are living microorganisms that provide health benefits such as improving digestive health and preventing infectious diarrhea, irritable bowel syndrome, and inflammatory bowel disease of hosts (O’Callaghan and van Sinderen, 2016; Floch, 2018; Shehata et al., 2019). Health benefits of probiotics are species- or strain- specific. Not all lactic acid bacteria are considered as probiotics (Pinto-Sanchez et al., 2017). *Bifidobacterium* is one important member of probiotics that has benefits such as anti-cancer effects (Inoue et al., 2009) and reducing cholesterol level (Zhang et al., 2016) for the host. *Bifidobacterium* is Gram-positive, non-motile, and catalase-negative lactic acid bacterium that survives in the intestine of human. *Bifidobacterium* species in human gut microbiota include *Bifidobacterium longum*, *Bifidobacterium bifidum*, *Bifidobacterium breve*, *Bifidobacterium adolescentis*, *Bifidobacterium dentium*, and *Bifidobacterium pseudocatenulatum* (Junick and Blaut, 2012). *Bifidobacterium pseudolongum* and *Bifidobacterium thermophilum* previously considered to be of animal origin have been isolated from baby feces and human adults, respectively (von Ah et al., 2007; Turroni et al., 2009; Junick and Blaut, 2012).

*Bifidobacterium* species have universally different functions according to subspecies. For instance, *Bifidobacterium animalis* subsp. *lactis* has a strong anti-inflammatory effect to improve the immune system (Weizman et al., 2005), whereas *B. animalis* subsp. *animalis* cannot grow in milk (Masco et al., 2004). *B. longum* also has different types of glycolytic enzymes according to its subspecies (LoCascio et al., 2010; Lewis et al., 2016). Hence, differentiating *Bifidobacterium* subspecies is necessary. Furthermore, presenting correct species in probiotic products is critical for providing correct information to consumers and claiming health benefits of the product (Shehata et al., 2019). Recently, some studies have shown mislabeling issues such as absence of some species, inaccurate taxonomy information, and undeclared species (Lewis et al., 2016; Morovic et al., 2016) of commercial probiotic products. However, there is no reliable detection method to distinguish different species and subspecies of *Bifidobacterium*.

Polymerase chain reaction (PCR)-based methods have been widely used to detect bacterial strains in probiotics, dairy products, meat products, and seafood (Binetti et al., 2008; Cammà et al., 2012; Kim et al., 2019). In particular, the 16S rRNA gene has been used as a useful target gene for bacterial identification. However, the resolution of this gene among closely related species is low (Junick and Blaut, 2012). To differentiate *Bifidobacterium* species, more distinguishable identification markers need to be found because 16S rRNA genes of *Bifidobacterium* species share high similarities (mean, 95%) (Ventura et al., 2006; Junick and Blaut, 2012). Housekeeping genes such as *recA* (Ventura and Zink, 2003), *tuf* (Ventura and Zink, 2003), *atpD* (Ventura et al., 2004a), *groEL* (Zhu et al., 2003), and *groES* (Ventura et al., 2004b) have been used as alternative genetic markers for the discrimination of *Bifidobacterium*. Although these genes have been demonstrated to have a relatively higher resolution than 16S rRNA gene, similar species and subspecies are still indistinguishable. Thus, those genes can only be applied to limited species (Lawley et al., 2017).

Whole-genome sequencing (WGS) is a powerful method for identifying unique genes through bioinformatics (Chen et al., 2010; Mellmann et al., 2017). Comparative genomics has been performed for pathogenic bacteria and lactic acid bacteria using various algorithms (Lugli et al., 2017; Zhang et al., 2019). But, studies on development of specific primers of probiotic species based on comparative genomics have not been widely conducted. The objective of the present study was to develop a real-time PCR assay using comparative genomics known to be able to detect highly specific genetic markers and bacterial strains very quickly. A brief description of the method is as follows: specific genetic markers were selected using comparative genomics from 210 *Bifidobacterium* genomes, and species- or subspecies-specific primers were designed based on identified markers. Real-time PCR assay was then applied for quantitative identification of 22 *Bifidobacterium* species, which is mainly found in intestine of human and food samples such as probiotic or dairy products and difficult to differentiate by conventional methods. Furthermore, label claims of commercial probiotics and dairy products were verified using the developed real-time PCR assay.

## Materials and Equipment

### Bacterial Strains

Forty-one *Bifidobacterium* species or subspecies strains, 11 *Lactobacillus* species, 1 *Lactococcus* species, and 2 *Enterococcus* species obtained from Korean Agricultural Culture Collection (KACC, Jeonju, South Korea), Korean Collection for Type Cultures (KCTC, Daejeon, South Korea), and Korean Culture Center of Microorganisms (KCCM, Seoul, South Korea) were used to confirm the specificity of the developed real-time PCR (Table 1).

**TABLE 1:** List of strains used in this study.

Species	Strain number
*Bifidobacterium animalis* subsp. *animalis*	KACC 16637
*Bifidobacterium animalis* subsp. *lactis*	KACC 16638
*Bifidobacterium animalis* subsp. *lactis*	LI 001941
*Bifidobacterium animalis* subsp. *lactis*	LI 001942
*Bifidobacterium animalis* subsp. *lactis*	LI 000026
*Bifidobacterium animalis* subsp. *lactis*	LI 000004
*Bifidobacterium animalis* subsp. *lactis*	LI 000019
*Bifidobacterium animalis* subsp. *lactis*	LI 000062
*Bifidobacterium breve*	KACC 16639
*Bifidobacterium breve*	KCTC 3419
*Bifidobacterium breve*	LI 000070
*Bifidobacterium longum* subsp. *infantis*	KCTC 3249
*Bifidobacterium longum* subsp. *infantis*	LI 000033
*Bifidobacterium longum* subsp. *infantis*	LI 000261
*Bifidobacterium longum* subsp. *infantis*	LI 000262
*Bifidobacterium longum* subsp. *suis*	KACC 16649
*Bifidobacterium longum* subsp. *longum*	KCCM 11953
*Bifidobacterium longum* subsp. *longum*	LI 000175
*Bifidobacterium bifidum*	KCTC 3418
*Bifidobacterium bifidum*	KCTC 3440
*Bifidobacterium bifidum*	LI 000058
*Bifidobacterium bifidum*	LI 000061
*Bifidobacterium bifidum*	LI 000063
*Bifidobacterium gallicum*	KACC 16645
*Bifidobacterium thermacidophilum*	KACC 16653
*Bifidobacterium thermacidophilum*	KACC 16674
*Bifidobacterium thermophilum*	KACC 20600
*Bifidobacterium coryneforme*	KACC 16642
*Bifidobacterium asteroides*	KACC 16635
*Bifidobacterium adolescentis*	KACC 16634
*Bifidobacterium pseudolongum*	KACC 16667
*Bifidobacterium pseudolongum*	KACC 16666
*Bifidobacterium cuniculi*	KACC 16643
*Bifidobacterium gallinarum*	KACC 16646
*Bifidobacterium scardovii*	KACC 16672
*Bifidobacterium pseudocatenulatum*	KCTC 3223
*Bifidobacterium angulatum*	KCTC 3236
*Bifidobacterium dentium*	KACC 16644
*Bifidobacterium tsurumiense*	KACC 16654
*Bifidobacterium catenulatum*	KACC 16640
*Bifidobacterium catenulatum*	KACC 16648
*Lactobacillus gasseri*	KCTC 3163
*Lactobacillus rhamnosus*	KCTC 3237
*Lactobacillus casei*	KACC 12413
*Lactobacillus delbrueckii*	KACC 12420
*Lactobacillus acidophilus*	KACC 12419
*Lactobacillus helveticus*	KACC 12418
*Lactobacillus fermentum*	KACC 11441
*Lactobacillus paracasei*	KACC 12427
*Lactobacillus plantarum*	KACC 11451
*Lactobacillus reuteri*	KCTC 3594
*Lactobacillus salivarius*	KCTC 3600
*Lactococcus lactis*	KACC 19376
*Enterococcus faecium*	KCTC 13225
*Enterococcus faecalis*	KCTC 3206

*KACC, the Korean Agricultural Culture Collection; LI, the Laboratory Isolate; KCTC, the Korean Collection for Type Cultures; KCCM, the Korean Culture Center of Microorganisms.*

### Equipment and Software

Anvi’o, Bacterial Pan Genome Analysis pipeline (BPGA), USEARCH, and Basic Local Alignment Search Tool (BLAST) software were used for comparative genomics to select specific genetic genes for *Bifidobacterium* species or subspecies. 7500 Real-time PCR System (Applied Biosystems, Foster City, CA, United States) and 7500 software were used for the specificity and accuracy of species- or subspecies-specific primers.

## Methods

### Cultivation and Genomic DNA Extraction of *Bifidobacterium* Strains

*Bifidobacterium* strains were cultured in Bifidobacterium broth (MB cell, Seoul, South Korea) and BL broth (MB cell, Seoul, South Korea) at 37°C for 48 h under anaerobic condition. Other lactic acid bacterial strains were cultured in MRS broth (Difco, Becton Dickinson, Sparks, MD, United States) at 30°C for 48 h under anaerobic condition (Kim et al., 2020). All strains were stored in 30% (v/v) glycerol (Bioshop, Burlington, ON, Canada) at −80°C until use. To extract genomic DNA, all cultured bacterial cells were collected by centrifugation at 16,200 × *g* for 3 min. DNeasy Blood and Tissue kit (Qiagen, Hilden, Germany) was used to extract total genomic DNAs from all strains following the manufacturer’s protocol for Gram-positive bacteria. Purity and concentration of extracted bacterial DNA were measured using a MaestroNano^®^ spectrophotometer (Maestrogen, Las Vegas, NV, United States).

### Genomic DNA Extraction of Commercial Products

Commercial products used in this study are listed in Table 2. These products were classified from A1 to A26 for probiotic products and from B1 to B7 for dairy products. Twenty-six probiotic products and 7 dairy products were purchased from markets worldwide (South Korea: 16, United States: 7, Canada: 8, United Kingdom: 1, Italy: 1). These probiotic products included 18 capsules, 7 powders, and 1 chewable. Total genomic DNAs of probiotic products were extracted according to a previous study (Kim et al., 2017). One-hundred milligrams of probiotic product were aliquoted and dissolved in 300 μL of lysis buffer following the manufacture’s instruction (DNeasy Blood and Tissue kit, Qiagen). Purity and concentration of extracted probiotic DNAs were measured as previously mentioned.

**TABLE 2:** Type, form, and country of purchase in probiotic and dairy products.

Products	Type	Form	Country
A1	Probiotic product	Capsules	Canada
A2	Probiotic product	Chewable	South Korea
A3	Probiotic product	Capsules	United States
A4	Probiotic product	Capsules	United States
A5	Probiotic product	Capsules	United States
A6	Probiotic product	Powder	South Korea
A7	Probiotic product	Capsules	Canada
A8	Probiotic product	Powder	South Korea
A9	Probiotic product	Capsules	United States
A10	Probiotic product	Capsules	Canada
A11	Probiotic product	Capsules	Canada
A12	Probiotic product	Capsules	Canada
A13	Probiotic product	Capsules	United States
A14	Probiotic product	Powder	South Korea
A15	Probiotic product	Powder	South Korea
A16	Probiotic product	Powder	South Korea
A17	Probiotic product	Capsules	United Kingdom
A18	Probiotic product	Capsules	United States
A19	Probiotic product	Powder	South Korea
A20	Probiotic product	Capsules	Italy
A21	Probiotic product	Capsules	Canada
A22	Probiotic product	Capsules	United States
A23	Probiotic product	Capsules	Canada
A24	Probiotic product	Capsules	South Korea
A25	Probiotic product	Capsules	Canada
A26	Probiotic product	Powder	South Korea
B1	Dairy product	Yogurt	South Korea
B2	Dairy product	Yogurt	South Korea
B3	Dairy product	Yogurt	South Korea
B4	Dairy product	Yogurt	South Korea
B5	Dairy product	Yogurt	South Korea
B6	Dairy product	Yogurt	South Korea
B7	Dairy product	Yogurt	South Korea

### Comparative Genomic Analysis of *Bifidobacterium* Species or Subspecies

All *Bifidobacterium* genome sequences were downloaded from the National Center for Biotechnology Information (NCBI)^1^, including 110 complete genomes, 52 scaffolds, and 31 contigs (Supplementary Table S1). To avoid drawing incorrect conclusions from the genomic analysis due to mislabeled genomes, a total of 210 *Bifidobacterium* genomes were evaluated using phylogenetic trees based on pan and core genes. A phylogenetic tree based on the pan-genome was constructed using Anvi’o version 6.0 publically available software according to the workflow for pan-genomics (Eren et al., 2015). Genome sequences obtained from NCBI were stored in Anvi’o storage for genomes to build a genome database. Pan-genome analysis was performed with the genome database. A phylogenetic tree was constructed according to pan gene cluster frequencies. Also, a phylogenetic tree based on core genes was constructed using BPGA version 1.3. The core genes were aligned using MUSCLE in BPGA, and a neighbor-joining phylogenetic tree was constructed. To select *Bifidobacterium* species- or subspecies-specific genetic markers, the core genome common to each species or subspecies was constructed. Core genomes were then compared to explore candidate genetic markers using BPGA version 1.3 with default identity value (Chaudhari et al., 2016). Final candidates for species- or subspecies-specific genetic markers were verified using BLAST against 57,122,612 sequences, including sequences of other lactic acid bacteria. Then 22 genetic markers and 210 genome sequences were aligned with UBLAST algorithm with USEARCH version 9.0 (Edgar, 2010). The alignment of genetic markers to genomes is shown in a heatmap (Figure 2). Also, the presence/absence of genes is easily skewed when the selected genetic marker is variable, so for all genetic markers their locations were verified, such as whether they are located in prophage genomes and plasmids or are really part of the core genome of that species using PlasmidFinder version 2.1 and BLAST analysis. Species- and subspecies-specific primers were designed based on selected genetic markers using Primer Designer (Scientific and Education Software, Chapel Hill, NC, United States). All 22 primer pairs were developed to be less than 200 bp in size to increase the amplification efficiency (Kim et al., 2020) suitable for the application of processed food products. All primers were synthesized by Bionics (Seoul, South Korea).

### Specificity and Standard Quantification Using Real-Time PCR Assay

To confirm the specificity of designed primers, real-time PCR was performed for 41 *Bifidobacterium* strains and 14 non-*Bifidobacterium* strains using a 7500 Real-time PCR System (Applied Biosystems, Foster City, CA, United States). The reaction mixture consisted of 10 μL of 2 × Thunderbird SYBR^®^ qPCR Mix (Toyobo, Osaka, Japan), 20 ng of template DNA, 0.5 μM of each primer pair, and deionized-distilled water to have a total volume of 20 μL. Each target was amplified with the following conditions: initiation at 95°C for 2 min for one single step, followed by 35 cycles at 95°C for 5 s and 60°C for 30 s. A melting curve was generated in the range of 95°C for 15 s, 60°C for 1 min, 95°C for 30 s, and 60°C for 15 s. Standard curve was obtained according to previously reported methods (Gómez-Rojo et al., 2015; Kim et al., 2020). Briefly, *Bifidobacterium* strains at 8 × 10^5^ to 8 × 10^9^ CFU/mL as determined by plate counting on Bifidobacterium agar (MB cell, Seoul, South Korea) were subjected to DNA extraction. Amplification was repeated three times. Standard curves for quantification were obtained by plotting *C*_t_ values against the number of bacteria per reaction (log CFU/mL). Results of real-time PCR assay were evaluated using 7500 software v2.0.6 (Applied Biosystems).

### Application of the Developed Real-Time PCR Assay for Probiotic Products

Probiotic products were monitored to detect 22 *Bifidobacterium* species or subspecies using the real-time PCR developed in this study (Supplementary Figure S1). For the application, 20 ng of DNA from each probiotic product was added to each well of a 96-well plate containing 2 × Thunderbird SYBR^®^ qPCR Mix (Toyobo, Osaka, Japan) and the species- or subspecies-specific primers. Real-time PCR was performed using a 7500 Real-Time PCR system (Applied Biosystems). PCR conditions were the same as indicated above in the “Specificity and Standard Quantification using Real-Time PCR Assay” section.

## Results

### Comparative Genomic Analysis of *Bifidobacterium*

**FIGURE 1:**
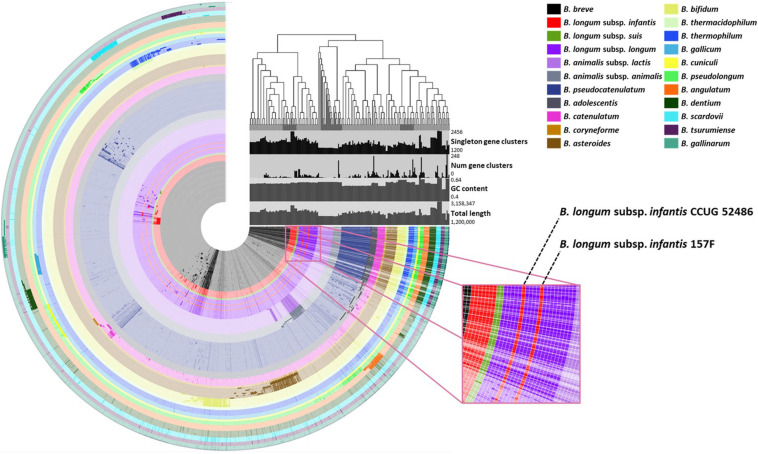
Pan-genomic phylogenetic tree of the *Bifidobacterium*. The figure shows that each ring represents *Bifidobacterium* genome and each layer displays the pan-genome distribution. The dark and bright colors of each ring indicate the presence and absence of core genes, respectively.

Species- or subspecies-specific genetic markers were selected using comparative genomic analysis for 210 *Bifidobacterium* genomes. Candidate genetic markers for targets were selected by comparing core genomes with non-target pan-genome. To select specific genetic markers for the target, candidate genetic markers were blasted against 57,122,612 sequences, including sequences of other lactic acid bacteria. A phylogenetic tree was constructed based on the pan-genome for *Bifidobacterium*. Phylogeny showed that most genomes (*n* = 208) shared the same lineage according to their species or subspecies type (Figure 1). In contrast, *B. longum* subsp. *infantis* CCUG 52486 and 157F were more closely related to *B. longum* subsp. *longum* group than to *B. longum* subsp. *infantis*. The phylogenetic tree constructed by core genomes also showed the same clusters, where these two *B. longum* subsp. *infantis* genomes were clustered into *B. longum* subsp. *longum* (Supplementary Figure S2).

**FIGURE 2:**
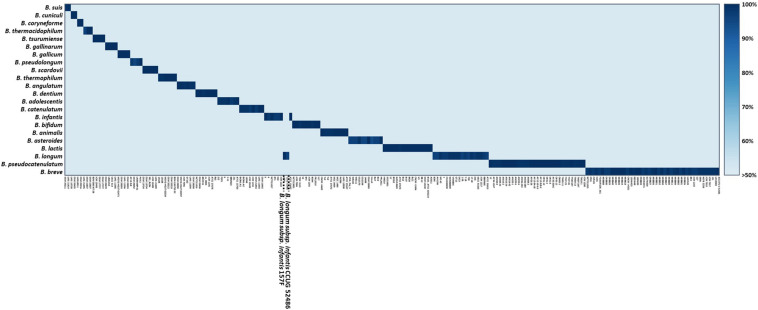
Alignment of *Bifidobacterium* genome and species- or subspecies-specific genetic markers.

A total of 372,743 genes yielded a pan-genome of 21,669 genes. The core genome had 250 genes. The accessory genome had 15,429 genes. The unique genome had 7,170 genes. The unique genome was divided into genetic markers common to the same species or subspecies. The specificity of identified genetic markers was confirmed by BLAST. Most of these genomes (208/210, 99.05%) shared 90–100% sequence identities within genetic markers of the same species or subspecies and 0–50% sequence identities against other species. Information of these genes is shown in Table 3. These identified genetic markers shared more than 90% sequence identities against each target genome except two *B. longum* subsp. *infantis* strains. These two strains were classified into *B. longum* subsp. *longum* according to our pan-genome analysis (Figure 2). The genetic marker for *B. longum* subsp. *infantis* such as ABC transporter permease (accession no. WP012576966.1) was present in 7 out of 9 strains (except CCUG 52486 and 157F). Instead, *B. longum* subsp. *infantis* CCUG 52486 and 157F had a bacterial Ig-like domain-containing protein (WP013141462.1), a genetic marker for *B. longum* subsp. *longum*. We confirmed that in these two *B. longum* subsp. *infantis* genomes, the genetic marker of *B. longum* subsp. *longum* was not present in their plasmids but on the chromosome, by blasting the contigs against the reported plasmid sequences. As well as, all genetic markers identified in this study were not located in plasmids or phage proteins and present in chromosome, meaning that these genetic markers are not variable and are part of the core genome. Based on these results, species- or subspecies-specific primers were designed and used for further studies (Table 4).

**TABLE 3:** The accession number and information of species- or subspecies-specific genetic markers.

Target species	Species- or subspecies-specific genetic markers	Accession no.
*B. animalis* subsp. *animalis*	Hypothetical protein	AFI62648.1
*B. animalis* subsp. *lactis*	Sel1 repeat family protein	WP004218390.1
*B. breve*	Serine hydrolase	WP014483379.1
*B. longum* subsp. *infantis*	ABC transporter permease	WP012576966.1
*B. longum* subsp. *suis*	Glycosyl hydrolase, BNR repeat-containing protein	KFI72947.1
*B. longum* subsp. *longum*	Bacterial Ig-like domain-containing protein	WP013141462.1
*B. bifidum*	Conserved hypothetical protein containing Ig-like domain	ADO53681.1
*B. gallicum*	Adhesin isopeptide-forming adherence domain-containing protein	WP052295095.1
*B. thermacidophilum*	Hypothetical protein	KFI99790.1
*B. thermophilum*	RelA/SpoT domain containing protein	AGH40345.1
*B. coryneforme*	Hypothetical protein	WP038459169.1
*B. asteroides*	Conserved repeat domain protein with Cna protein B-type	AFU70840.1
*B. adolescentis*	MFS transporter	WP011743138.1
*B. pseudolongum*	Hypothetical protein	WP022857512.1
*B. cuniculi*	Hypothetical protein	WP033518587.1
*B. gallinarum*	ATP-binding protein	WP081929610.1
*B. scardovii*	DNA helicase	KFI95242.1
*B. pseudocatenulatum*	Hypothetical protein	WP004223713.1
*B. angulatum*	Type 2 lantipeptide synthetase LanM	WP052946496.1
*B. dentium*	Cna B-type domain-containing protein	WP003837636.1
*B. tsurumiense*	BspA family leucine-rich repeat surface protein	WP026642738.1
*B. catenulatum*	Transcriptional regulator	WP003833517.1

**TABLE 4:** Primer information used in this study.

Target species	Primer name	Sequence (5′-3′)	Size (bp)
*B. animalis* subsp. *animalis*	Animalis-F	CAG ACC TCG CCG ATG AGC TA	110
	Animalis-R	ATA TCC GGC TTG ATC ACC TG	
*B. animalis* subsp. *lactis*	Lactis-F	ACC TCA CCA ATC CGC TGT TC	137
	Lactis-R	GAT CCG CAT GGT GGA ACT CT	
*B. breve*	Breve-F	TCA TCA CGG CAA GGT CAA GA	111
	Breve-R	GGC CAG AAC AGC TGG AAC AA	
*B. longum* subsp. *infantis*	Infantis-F	ATG ATG CGC TGC CAC TGT TA	132
	Infantis-R	CGG TGA GCG TCA ATG TAT CT	
*B. longum* subsp. *suis*	Suis-F	CAA GCC GGA TAT CGT CTT CG	130
	Suis-R	GAG GAT CGT GCC ATG CTG TC	
*B. longum* subsp. *longum*	Longum-F	GTG TGG ATT ACC TGC CTA CC	179
	Longum-R	GTC GCC AAC CTT GAC CAC TT	
*B. bifidum*	Bifidum-F	CTG GCA GCC GTG ACA CTA CT	102
	Bifidum-R	TGA ACT GGC CGT TAC GGT CT	
*B. gallicum*	Gallicum-F	TCA CCA TCA CCA CCT CAC	182
	Gallicum-R	GTT CCA TTG TTC CCA TCC C	
*B. thermacidophilum*	Thermacidophilum-F	CGT TAG AAC AGC GCC AAC AG	116
	Thermacidophilum-R	GCC GGC ATA TTC ATC GAG TC	
*B. thermophilum*	Thermophilum-F	CCG ATG CCG ATA CAG TTC AA	109
	Thermophilum-R	TGT CAT CCG ACG CTT CAA GA	
*B. coryneforme*	Coryneforme-F	TAA ATT CGT CCC CGC TTT GC	144
	Coryneforme-R	TCC TCA TCC TCC TCC ATA ACC	
*B. asteroides*	Asteroides-F	GCC GTG GTC ACC ACA CTA TC	108
	Asteroides-R	GCG CAC TAT GTC ATT GTC TG	
*B. adolescentis*	Adolescentis-F	GCT GAT ATC TGC GCT GTA CC	135
	Adolescentis-R	AAA CCA CCC AGT AGT CCT CC	
*B. pseudolongum*	Pseudolongum-F	CAA GGC CAT CAA CTG GTT CA	120
	Pseudolongum-R	ACG TCG TGC TGC TCG AAT GT	
*B. cuniculi*	Cuniculi-F	TGA AGG AAA CAC CGC CAA TC	127
	Cuniculi-R	ACC TCC CTC TGA GCC TTG AC	
*B. gallinarum*	Gallinarum-F	CGA CGA AAC ATT ACG CAT CC	163
	Gallinarum-R	ATG AAA TCC ACT TCG CCA CC	
*B. scardovii*	Scardovii-F	CGC AGG CAC TCG CTG TAC TA	102
	Scardovii-R	GGC GTA ACG TCT CAG TAT CA	
*B. pseudocatenulatum*	Pseudocatenulatum-F	ACC TAC GAT TTC TCC CTC TCC	173
	Pseudocatenulatum-R	CTC CAG CAA AGC CAA CGA AC	
*B. angulatum*	Angulatum-F	TGC GGA TAC CAT CGA AGA AC	101
	Angulatum-R	TTC GCG ACA TCC ATT GAC TG	
*B. dentium*	Dentium-F	GCG ACC GCT TCC ATC ATT AT	123
	Dentium-R	GGA GAT GCC GTC CTT AGA TT	
*B. tsurumiense*	Tsurumiense-F	TGC GGT TCA ACC AAG CTT AC	167
	Tsurumiense-R	TCG TCG TCA CCA GAT TCT TC	
*B. catenulatum*	Catenulatum-F	CGC CAA CGC AGT AGT GCA TA	106
	Catenulatum-R	TAG GCC ACC TGG ATT CGA TA	

### Specificity and Quantification of the Developed Real-Time PCR Assay

**FIGURE 3:**
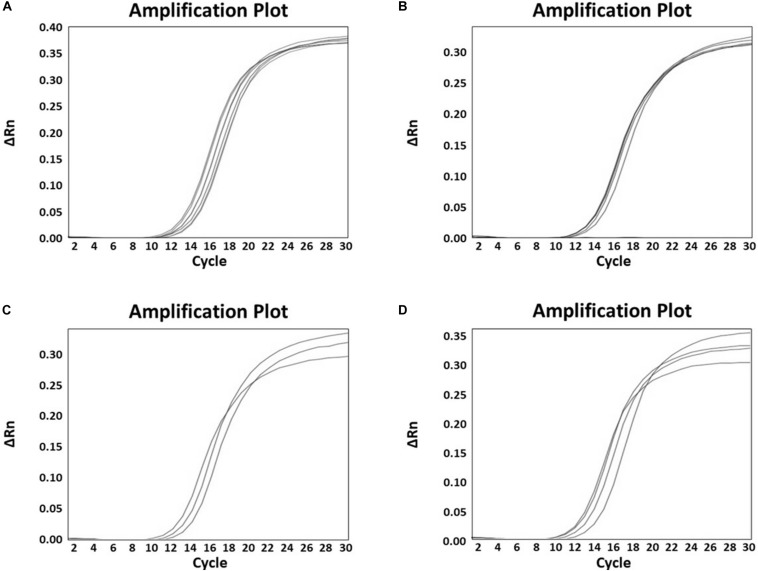
Specificity of species- and subspecies-specific primer pairs which were mainly used in probiotic products against 41 strains. **(A)** Specificity of *B. animalis* subsp. *lactis* specific primer pair, amplification curve: *B. animalis* subsp. *lactis* KACC 16638, LI 001941, LI 001942, LI 000026, LI 000004, LI 000019, and LI 000062; **(B)** Specificity of *Bifidobacterium bifidum* specific primer pair, amplification curve: *B. bifidum* KCTC 3418, KCTC 3440, LI 000058, LI 000061, and LI 000063; **(C)** Specificity of *Bifidobacterium breve* specific primer pair, amplification curve: *B. breve* KACC 16639, KCTC 3419, and LI 000070; **(D)** Specificity of *B. longum* subsp. *infantis* specific primer pair, amplification curve: *B. longum* subsp. *infantis* KCTC 3249, LI 000033, LI 000261, and LI 000262.

**FIGURE 4:**
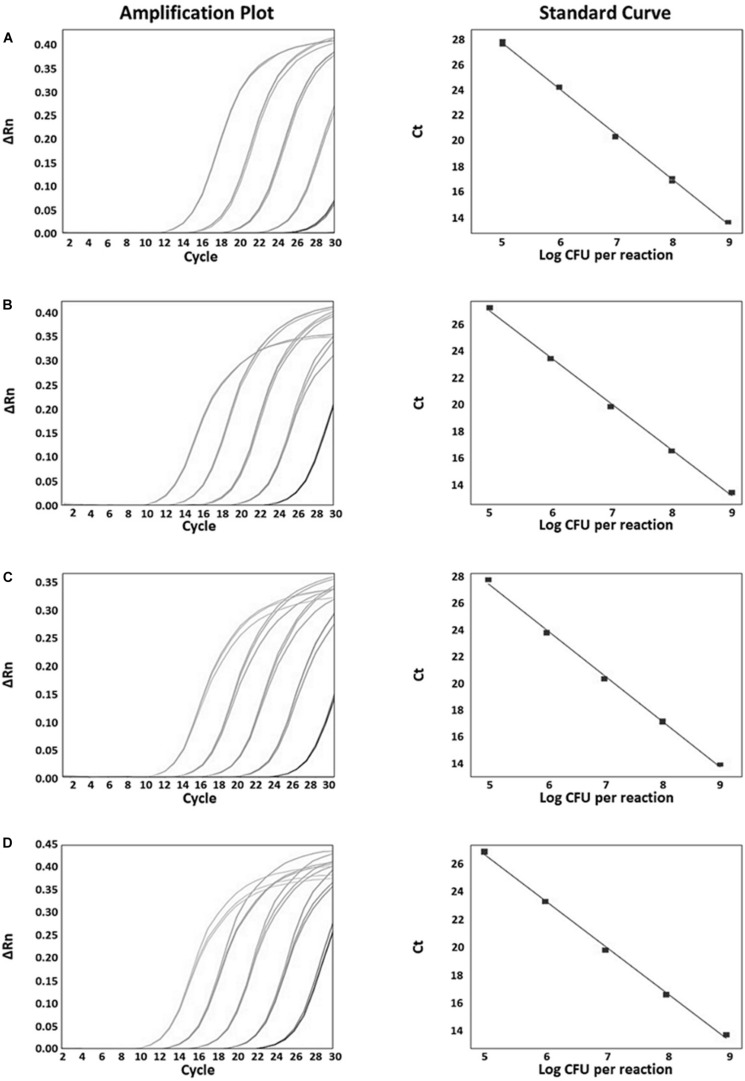
Real-time PCR amplification plots and standard curves which were mainly used in probiotic products for quantitative evaluation. **(A)** *B. animalis* subsp. *lactis* amplification plot (left), standard curve between 20 and 0.002 ng (*y* = –3.564*x* + 18.03, *R*^2^ = 0.998, Eff% = 90.788, right); **(B)** *B. bifidum* amplification plot (left), standard curve (*y* = –3.438*x* + 17.713, *R*^2^ = 0.998, Eff% = 95.359, right); **(C)** *B. breve* amplification plot (left), standard curve (*y* = –3.448*x* + 18.169, *R*^2^ = 0.998, Eff% = 94.987, right); **(D)** *B. longum* subsp. *infantis* amplification plot (left), standard curve (*y* = –3.312*x* + 17.727, *R*^2^ = 0.998, Eff% = 100.424, right).

The specificity of the developed real-time PCR assay was confirmed with 41 *Bifidobacterium* strains and 14 non-*Bifidobacterium* strains. As a result, all primer sets specific for each *Bifidobacterium* species/subspecies *in silico* showed detectable amplicons, with *C*_t_ values between 11 and 16 against target strains, whereas those from all non-targets did not generate any positive signal (Figure 3 and Supplementary Table S2). To quantify the number of bacteria and to confirm the accuracy of real-time PCR, a standard curve was obtained using template DNA of *Bifidobacterium* at a range of 8 × 10^5^ to 8 × 10^9^ CFU/mL in triplicates. This range included the number of bacteria labeled on probiotic products used. Slope for standard curves of *B. animalis* subsp. *lactis*, *B. bifidum*, *B. breve*, and *B. longum* subsp. *infantis* mainly used in probiotic products were −3.499, −3.134, −3.275, and −3.552, respectively. All *R*^2^ values (correlation coefficients) were ≥ 0.997 (Figure 4). Results of the slope, *R*^2^ value, and efficiency of remaining primers are shown in Supplementary Table S3. According to the efficiency of quantitative real-time PCR, *R*^2^ values ≥ 0.98 are considered as reliable (Broeders et al., 2014). Thus, the real-time PCR developed in this study was confirmed to be highly accurate and efficient.

### Monitoring of Probiotic and Dairy Products Using the Real-Time PCR Developed

Commercially available probiotic and dairy products were used to verify whether the real-time PCR developed in this study could be applicable to quantify and identify probiotics in food products (Supplementary Figure S1). A total of 33 commercial probiotic and dairy products containing *Bifidobacterium* were monitored. Obtained results were compared with product label claims. Results of 21 products were identical to their label claims. In particular, probiotic strains of eight products that were only labeled at the species level such as *B. longum* and *B. animalis* were able to be analyzed up to subspecies level using our real-time PCR assay (Table 5). For the remaining four products (B4 to B7) labeled as “Lactic acid bacteria or *Bifidus*,” this real-time PCR assay was able to detect *Bifidobacterium* at the subspecies level. Based on the standard quantitative curve for each *Bifidobacterium* species or subspecies obtained by plotting *C*_t_ values against the number of bacteria per reaction, the number of *Bifidobacterium* species or subspecies present in the food products was estimated to be within the range of 8 × 10^5^ to 8 × 10^9^ CFU/mL. Thus, the real-time PCR method developed in this study could accurately detect and quantify *Bifidobacterium* strains contained in probiotic and dairy products at species level and subspecies level.

**TABLE 5:** Monitoring of commercial probiotic and dairy products for the verification of the developed 96-well plate.

Products	Label claim	Detected species or subspecies
A1	*B. longum*	*B. longum* subsp. *longum*
A2	*B. bifidum*, *B. longum*	*B. bifidum*, *B. longum* subsp. *longum*
A3	*B. animalis* subsp. *lactis*	*B. animalis* subsp. *lactis*
A4	*B. animalis* subsp. *lactis*	*B. animalis* subsp. *lactis*
A5	*B. animalis* subsp. *lactis*	*B. animalis* subsp. *lactis*
A6	*B. animalis* subsp. *lactis*	*B. animalis* subsp. *lactis*
A7	*B. bifidum*, *B. breve*, *B. longum*	*B. bifidum*, *B. breve*, *B. longum* subsp. *longum*
A8	*B. animalis* subsp. *lactis*, *B. bifidum*	*B. animalis* subsp. *lactis*, *B. bifidum*
A9	*B. animalis* subsp. *lactis*, *B. bifidum*	*B. animalis* subsp. *lactis*, *B. bifidum*
A10	*B. breve*, *B. longum* subsp. *longum*	*B. breve*, *B. longum* subsp. *longum*
A11	*B. animalis* subsp. *lactis*, *B. bifidum*, *B. breve*	*B. animalis* subsp. *lactis*, *B. bifidum*, *B. breve*
A12	*B. breve*, *B. longum*, *B. longum* subsp. *infantis*	*B. breve*, *B. longum* subsp. *longum*, *B. longum* subsp. *infantis*
A13	*B. animalis* subsp. *lactis*, *B. breve*, *B. longum*	*B. animalis* subsp. *lactis*, *B. breve*, *B. longum* subsp. *longum*
A14	*B. animalis* subsp. *lactis*, *B. bifidum*, *B. breve*	*B. animalis* subsp. *lactis*, *B. bifidum*, *B. breve*
A15	*B. animalis* subsp. *lactis*, *B. bifidum*, *B. breve*	*B. animalis* subsp. *lactis*, *B. bifidum*, *B. breve*
A16	*B. animalis* subsp. *lactis*, *B. bifidum*, *B. breve*	*B. animalis* subsp. *lactis*, *B. bifidum*, *B. breve*
A17	*B. animalis* subsp. *lactis*, *B. bifidum*, *B. breve*, *B. longum*	*B. animalis* subsp. *lactis*, *B. bifidum*, *B. breve*, *B. longum* subsp. *longum*
A18	*B. animalis* subsp. *lactis*, *B. bifidum*, *B. breve*, *B. longum*	*B. animalis* subsp. *lactis*, *B. bifidum*, *B. breve*, *B. longum* subsp. *longum*
A19	*B. animalis* subsp. *lactis*, *B. bifidum*, *B. breve*, *B. longum*	*B. animalis* subsp. *lactis*, *B. bifidum*, *B. breve*, *B. longum* subsp. *longum*
A20	*B. animalis* subsp. *lactis*, *B. bifidum*, *B. breve*, *B. longum*	*B. animalis* subsp. *lactis*, *B. bifidum*, *B. breve*, *B. longum* subsp. *longum*, *B. longum* subsp. *infantis*
A21	*B. animalis* subsp. *lactis*, *B. bifidum*, *B. breve*, *B. longum* subsp. *longum*	*B. animalis* subsp. *lactis*, *B. bifidum*, *B. breve*, *B. longum* subsp. *longum*
A22	*B. animalis* subsp. *lactis*, *B. bifidum*, *B. breve*, *B. longum* subsp. *longum*, *B. longum* subsp. *infantis*	*B. animalis* subsp. *lactis*, *B. bifidum*, *B. breve*, *B. longum* subsp. *longum*, *B. longum* subsp. *infantis*
A23	*B. animalis* subsp. *lactis*, *B. bifidum*, *B. breve*, *B. longum* subsp. *longum*, *B. longum* subsp. *infantis*	*B. animalis* subsp. *lactis*, *B. bifidum*, *B. breve*, *B. longum* subsp. *longum*, *B. longum* subsp. *infantis*
A24	*B. animalis* subsp. *lactis*, *B. bifidum*, *B. breve*, *B. longum* subsp. *longum*, *B. longum* subsp. *infantis*	*B. animalis* subsp. *lactis*, *B. bifidum*, *B. breve*, *B. longum* subsp. *longum*, *B. longum* subsp. *infantis*
A25	*B. animalis* subsp. *lactis*, *B. bifidum*, *B. breve*, *B. longum*, *B. longum* subsp. *infantis*	*B. animalis* subsp. *lactis*, *B. bifidum*, *B. breve*, *B. longum* subsp. *longum*, *B. longum* subsp. *infantis*
A26	*B. animalis* subsp. *lactis*, *B. bifidum*, *B. breve*, *B. longum*, *B. longum* subsp. *infantis*	*B. animalis* subsp. *lactis*, *B. bifidum*, *B. breve*, *B. longum* subsp. *longum*, *B. longum* subsp. *infantis*
B1	*B. animalis* subsp. *lactis*	*B. animalis* subsp. *lactis*
B2	*B. animalis* subsp. *lactis*	*B. animalis* subsp. *lactis*
B3	*B. animalis* subsp. *lactis*	*B. animalis* subsp. *lactis*
B4	*Bifidus*, *Lactic acid bacteria*	*B. animalis* subsp. *lactis*
B5	*Bifidus*, *Lactic acid bacteria*	*B. animalis* subsp. *lactis*, *B. longum* subsp. *longum*
B6	*Lactic acid bacteria*	*B. animalis* subsp. *lactis*
B7	*Lactic acid bacteria*	*B. animalis* subsp. *lactis*

## Discussion

*Bifidobacterium* subspecies (*B. animalis* subsp. *animalis* or *B. animalis* subsp. *lactis* and *B. longum* subsp. *longum* or *B. longum* subsp. *infantis*) are known to be similar to each other. However, these subspecies have different functions such as having ability to grow in milk or expressing enzymes (Masco et al., 2004). To distinguish these species or subspecies, previous studies have targeted marker genes such as 16S rRNA and *tuf* genes. However, it is difficult to distinguish subspecies by using these genes because of their highly similar sequences (Tannock et al., 2013; Kurakawa et al., 2015). Some researchers have screened specific genes through genomic analysis to distinguish *Bifidobacterium* subspecies. Lawley et al. (2017) have reported the identification of functional gene targets for the differentiation of *B. longum* subsp. *longum* and *B. longum* subsp. *infantis* based on comparative genomic analysis. However, these functional genes they identified showed some limitations. For example, *B. longum* subsp. *infantis* specific sialidase gene (accession no. ACJ53406.1) was limited to some *B. longum* subsp. *infantis* strains, but not all subspecies. It was also found to be present in *B. bifidum*. In addition, *B. longum* subsp. *longum* specific kinase gene (accession no. AAN24115.1) was present in many *Bifidobacterium* species such as *B. adolescentis* and *B. dentium*. Because of the limited number of genomes (*n* = 2) used in their analysis, these identified genes could not be applied to distinguish all *Bifidobacterium* species.

To overcome limitations of previous studies, we identified genetic markers with large-scale *Bifidobacterium* genome sequences (*n* = 210). All genetic markers obtained through comparative genomic analysis were confirmed to be specific by *in silico* analysis. We also confirmed that some genomes deposited in NCBI were misclassified. Previous studies have also reported that taxonomy information for similar species in the NCBI is incorrect (Kim et al., 2020). For *Bifidobacterium*, this is the first report to confirm the incorrect classification of genomes in NCBI. Inaccuracies of genomic information may contribute to difficulty in developing methods to distinguish *Bifidobacterium*. Our results suggest that *B. longum* subsp. *infantis* CCUG 52486 and 157F are *B. longum* subsp. *longum*.

The real-time PCR method developed in this study showed high specificity and accuracy. However, the limited information of some species, such as *Bifidobacterium coryneforme*, *Bifidobacterium cuniculi*, and *B. longum* subsp. *suis* were available in the NCBI (only one or two representatives), thus, we can only include the small number of genomes for those strains. This method was also successfully applied to monitoring of probiotic products. It correctly identified *Bifidobacterium* species contained in all products. We were also able to analyze these strains up to subspecies level labeled in probiotic products as *B. animalis* and *B. longum*, allowing us to better understand the presence of strains contained in probiotic products. A previous study (Patro et al., 2016) using shotgun next-generation sequencing has shown that nine out of ten probiotic products are consistent with their label claims. One product, which was misidentified, contained *B. longum* subsp. *longum* instead of *B. longum* subsp. *infantis*. They found that these strains were frequently mislabeled in other products (Patro et al., 2016). In another study (Lewis et al., 2016), 16 probiotic products containing *Bifidobacterium* were monitored by terminal-restriction fragment length polymorphism (T-RFLP) profiling. It was found that only one product was consistent with label claims (Lewis et al., 2016). Our assay can also distinguish between *B. longum* subsp. *longum* and *B. longum* subsp. *infantis*. The resolution of our method is comparable to shotgun sequencing. It is better than T-RFLP typing based on 16S rRNA gene. To provide more detailed information of commercial probiotic products to consumers, identifying and quantifying bacteria strains in food products is important. However, in this study, since quantification was performed on the DNA isolated from a culture, the concentration of *Bifidobacterium* may be underestimated when applied to a food matrix, as reported in previous studies (Kralik and Ricchi, 2017; Frentzel et al., 2018). Our real-time PCR assay can be used to differentiate and quantify multiple *Bifidobacterium* subspecies in food sample. The methods described in this study, such as the identification of genetic marker using pan-genome analysis and the design of specific primers using the selected genetic markers, can be applied to pathogenic bacteria in complex clinical samples and other bacterial strains as well.

## Conclusion

In conclusion, genetic markers were identified to distinguish different *Bifidobacterium* species and subspecies through comparative genomics based on their whole-genome sequences. Although *Bifidobacterium* species are commonly used in probiotic and dairy products, it is still difficult to distinguish all *Bifidobacterium* species by conventional detection methods. This study designed specific primers from these identified genetic markers. A real-time PCR assay was developed in this study to accurately and rapidly detect 22 *Bifidobacterium* in a single 96-well plate. The developed real-time PCR assay can be used to monitor commercial probiotic and dairy products. Our assay can also be used to verify the reliability of claims of probiotic and dairy products. Furthermore, it can be applied to identify *Bifidobacterium* communities in various food products and environmental samples.

## Data Availability Statement

The original contributions presented in the study are included in the article/Supplementary Material, further inquiries can be directed to the corresponding author.

## Author Contributions

H-BK, EK, S-MY, and H-YK designed the experiment. H-BK, EK, and SL performed the comparative genomic analysis. H-BK, EK, and S-MY confirmed primer specificity and performed application tests using real-time PCR. H-BK, EK, and M-JK prepared a draft manuscript. H-BK, EK, SL, and H-YK reviewed and edited the manuscript. All authors read and approved the final manuscript.

## Conflict of Interest

The authors declare that the research was conducted in the absence of any commercial or financial relationships that could be construed as a potential conflict of interest.

## Footnotes

1 https://www.ncbi.nlm.nih.gov/
