# Supplementary figures and images for: Development of Real-Time PCR Assay to Specifically Detect 22 Bifidobacterium Species and Subspecies Using Comparative Genomics
Source: Front Microbiol. 2020 Aug 28;11:2087. doi: 10.3389/fmicb.2020.02087 (PMC7493681; doi:10.3389/fmicb.2020.02087)

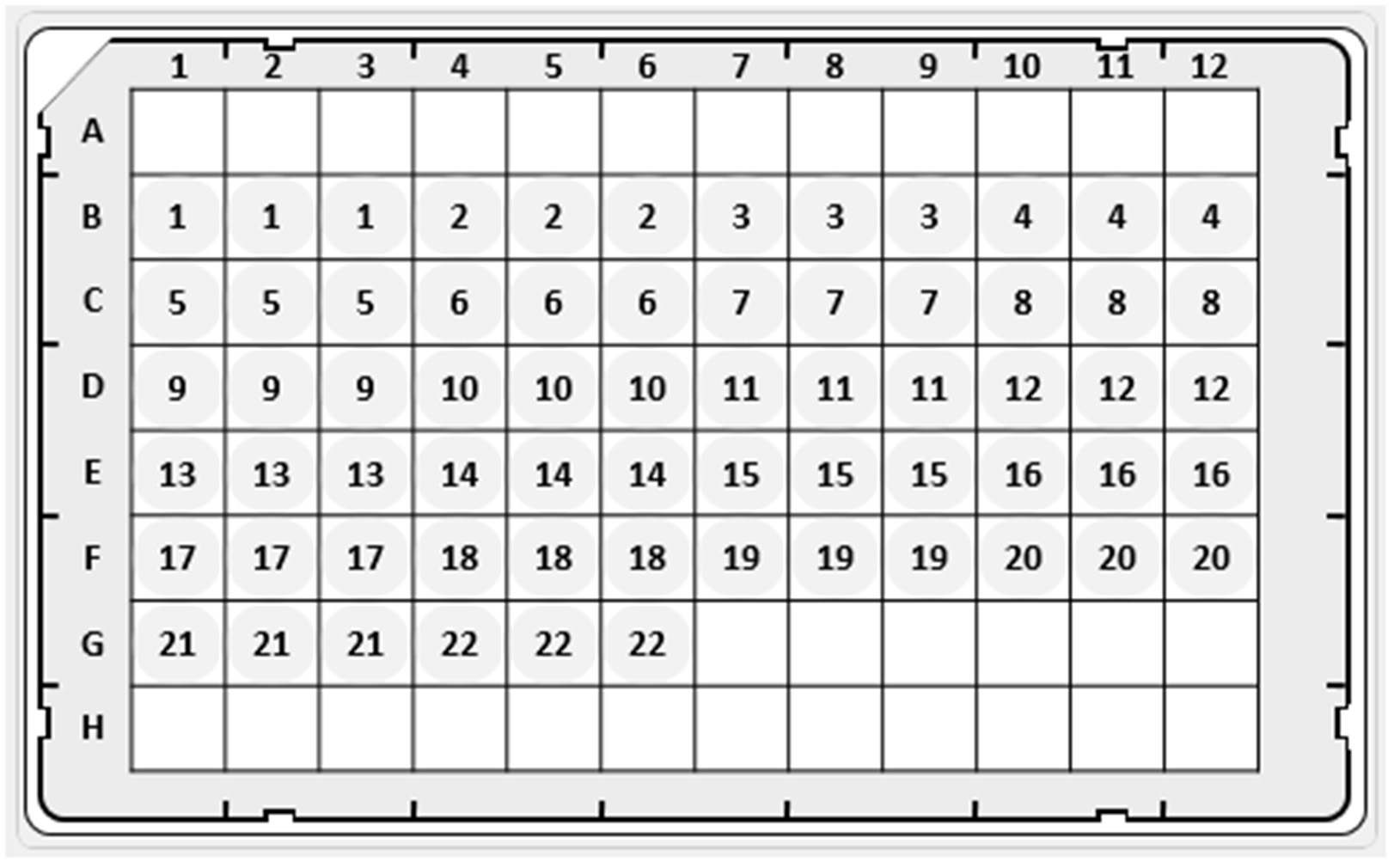

Supplement: Supplementary file 1 [file Image_1.JPEG]

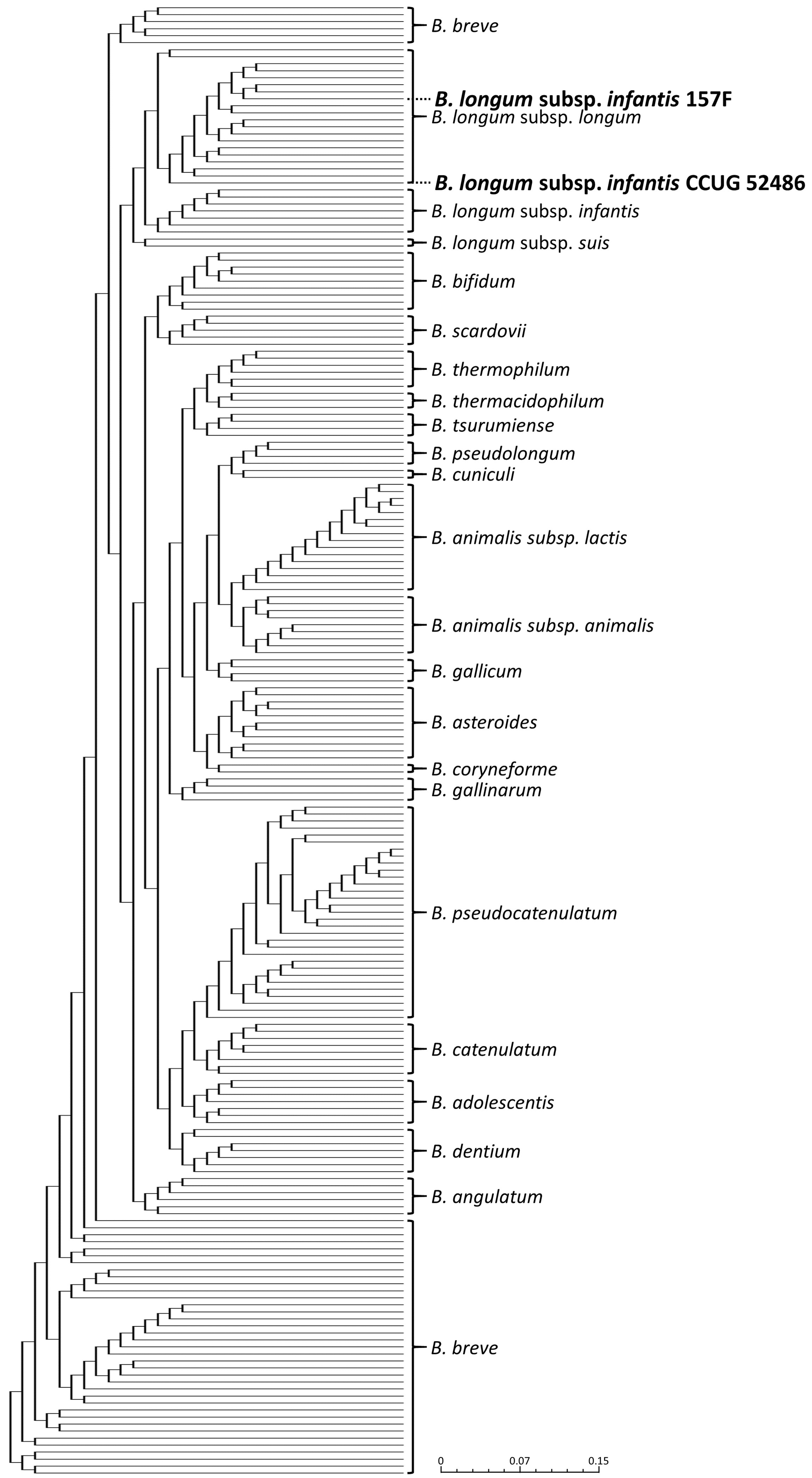

Supplement: Supplementary file 2 [file Image_2.JPEG]
